# Mitochondrial DNA Diversity in Large White Pigs in Russia

**DOI:** 10.3390/ani10081365

**Published:** 2020-08-06

**Authors:** Lyubov Getmantseva, Siroj Bakoev, Nekruz Bakoev, Tatyana Karpushkina, Olga Kostyunina

**Affiliations:** Federal Science Center for Animal Husbandry Named after Academy Member L.K. Ernst, Dubrovitsy 142132, Russia; siroj1@yandex.ru (S.B.); nekruz82@bk.ru (N.B.); tati.kriz@gmail.com (T.K.); kostolan@yandex.ru (O.K.)

**Keywords:** diversity, mtDNA, D-loop, pig, Large White, adaptation, climatic conditions

## Abstract

**Simple Summary:**

One of the most effective approaches to assessing populations is studying mitochondrial DNA (mtDNA) polymorphism. This work included studying variations in nucleotides in the field of a mitochondrial DNA D-loop in Large White pigs of various selections bred in the Russian Federation from 2000 to 2019 in order to define the presence of frequency distinctions of mtDNA haplotypes in Large White pigs of the Russian selection (created in the middle of the XX century) and the imported livestock of the Large White breed, which was the result of intensive selection work for increasing productive traits (growth rate, reproduction, forage costs, etc.). On the other hand, assessment of current Large White pigs from various farms of the Russian Federation is also of interest as they represent the selection of different international genetic centers. In addition, the aim of our work was to define the relationship between the allocation of mtDNA haplotypes frequencies with adaptation to different climatic conditions in Russian Large White pigs as far as breeding animals that were hardy to various environmental conditions had been one of the primary goals of that time.

**Abstract:**

The Large White pig is the most commonly raised commercial pig breed in the world. The aim of this work was to investigate D-loop mtDNA in Large White pigs (*n* = 402) of various selections bred in the Russian Federation from 2000 to 2019. The general sample consisted of three groups: Old (*n* = 78) (Russian selection, 2000–2010); Imp (*n* = 123) (imported to Russia in 2008–2014); New (*n* = 201) (2015–2019). The synthesized score (Fz) was calculated by analyzing the main PCA (principal component analysis components). An affiliation to Asian or European haplogroups was determined according to the NCBI (National Center for Biotechnology Information). In the study, we defined 46 polymorphic sites and 42 haplotypes. Significant distinctions between groups Old, Imp and New in frequencies of haplotypes and haplogroups were established. The distribution of Asian and European haplotypes in the groups was Old: 50%/50%, Imp: 43%/57%, New: 75%/25%, respectively. The variety of haplotypes and haplogroups in the pigs of the group New is related to the farms in which they breed. Haplotype frequencies significantly differ between the clusters Old_Center, Old_Siberia and Old_South. This study will provide information on the genetic diversity of Large White breed pigs. The results will be useful for the conservation and sustainable use of these resources.

## 1. Introduction

The Large White pig is the most commonly raised commercial pig breed around the world, due to the fact that Yorkshire pigs in the USA and Canada are direct descendants of the Large White line [1]. Today, a large majority of countries with developing or developed pig breeding have an imported Large White stock. The pedigree pigs of this breed are hardy, adaptable to climate, and can survive in various environments. Until the XIX century, local pigs occupied a prominent space in Europe. Various environmental conditions of certain regions impacted these animals [2]. Since the end of the XVIII century, breed improvement of local pigs began by creating Romanov, Oriental, and later by introducing Chinese pigs. Significant breeding work has led to creating the Large White breed.

Large White pigs were brought to Russia in the in the second half of the XIX century; however, during the wars in the beginning of the XX century, the larger half of the pedigree pigs were killed. In 1923–1931, a livestock of Large White pigs was imported from England for developing domestic pedigree pig breeding. Long-term breeding and the impact of climate and feeding changed the English type of Large White pigs and a new type of local (Russian) Large White breed was created which, at that time, was superior to the English one for many indicators [3]. Despite this, in the late XX to early XXI centuries, the pig livestock in Russia was almost completely replaced by imported pigs from leading breeding centers in Denmark, France, England, Holland, Ireland, etc.

One of the most effective approaches to assessing populations is studying mitochondrial DNA polymorphism. The mitochondrial genome is considered a mutation hotspot [4]. The rate of recorded mutations in mitochondrial genomes of animals is approximately 10 times higher than that in equivalent sequences in a nuclear genome [5]. For billions of years, the mitochondrial genome developed as organisms became adapted to the environment and selection pressure [6]. As a result, mutations became fixed, and various mitochondrial lines clustered in groups known as mtDNA haplogroups. mtDNA haplotypes determine certain distinctions because genetic variations in the mitochondrial genome are sensitive both to natural and artificial selection [7].

The mtDNA contains a non-coding (D-loop) sequence comprising regulatory sequences regulating both replication and transcription of the mtDNA [8]. Many studies of farm animals, including pigs, have shown the effectiveness of applying the D-loop as a diversity marker when studying the mitochondrial genome [9,10,11].

In connection with the above, a particular interest is represented by studies of pigs’ mtDNA bred at the breeding farms of the Russian Federation throughout the last two decades. This work includes studying variations of nucleotides in the field of a mtDNA D-loop in Large White pigs of various selections bred in the Russian Federation from 2000 to 2019 in order to define the presence of frequency distinctions of mtDNA haplotypes in Large White pigs of the Russian selection (created in the middle of the XX century) and the imported livestock of the Large White breed, which was the result of intensive selection work to increase productive traits (growth rate, reproduction, forage costs, etc.). On the other hand, assessment of current Large White pigs from various farms of the Russian Federation is also of interest as they represent the selection of different international genetic centers. The breeding lines belonging to these companies are the result of targeted selection based on their own technologies and know-how. In our study, we tried to define significant distinctions of genetic structure of mtDNA in different livestocks. In addition, the aim of our work was to define the relationship between the allocation of mtDNA haplotype frequencies with adaptation to different climatic conditions in Russian Large White pigs as far as breeding animals that were hardy to various environmental conditions had been one of the primary goals of that time. This research will allow us to obtain information about the genetic diversity of Large White pigs. The obtained results will contribute to the preservation and sustainable use of these resources and can also be used to improve the breed and create a Russian type of Large White breed.

## 2. Materials and Methods

The research was carried out on the basis of the Center for Collective Use of Scientific Equipment “Bioresources and Bioengineering of Agricultural Animals” of the L.K. Ernst Federal Research Center for Animal Husbandry [12]. Samples from the Unique Scientific Installation (UNU) “Bank of Genetic Materials of Animals and Birds” [13] were used for the work. For this collection, samples (tissue samples from the ear) were given by the owners of the breeding farms (according to their will). All methods were performed in accordance with the guidelines approved by the L.K. Ernst Federal Research Center for Animal Husbandry (Russia) and with the rules for conducting laboratory research (tests) in the implementation of veterinary control (supervision) approved by Council Decision Eurasian Economic Commission No. 80 (10 November 2017).

### 2.1. Animals

Research was conducted on Large White pigs of different selections. The general sample (*n* = 402) consisted of three groups. The first group (LW Old, *n* = 78) consisted of Large White pigs of Russian selection bred in 2000–2010. Breeding and selection of these animals since the 1950s have been fulfilled without adding import resources. The second group (LW Imp and York Imp, *n* = 123) was composed of Large White pigs and Yorkshire pigs delivered via import selection to the Russian Federation (RF) beginning in 2008–2014. The third group (LW New and York New) comprised Large White pigs and Yorkshire pigs, which are currently (2015–2019) kept at leading breeding farms in the Russian Federation. Table 1 represents all samples and the place and year of their selection.

### 2.2. PCR and Sequencing

Pig DNA was distinguished with a panel “K-Sorb-100” (“Synthol” Ltd.) according to manufacturer’s instructions. To amplificate a fragment of mtDNA D-loop, we conducted PCR (polymerase chain reaction), according to a standard procedure, using the following primers: F: 5′-TGC AAA CCA AAA CGC CAA GT-3′ and R: 3′-TTT TTG GGG TTT GGC AAG GC-5′. For primer design, we used NCBI Standard Nucleotide BLAST [14]. The 25 μL reaction mixture consisted of 3 μL of DNA sample (75 ng/μL), 5 μL of 10 × PCR-standard reaction buffer, 1 μL dNTP (1 mmol/L), 0.5 μL of each primer (20 μmol/L), and 0.5 μL Taq DNA polymerase. The amplification conditions were as follows: 95 °C for 4 min, followed by 33 cycles at 95 °C for 30 s, 64 °C for 30 s, and 72 °C for 45 s. The final step was 72 °C for 5 min. Visualization of the PCR products was made in the 2% agarose gel with the addition of ethidium bromide; the total length of the PCR fragment was 1046 bp. Specific PCR fragments were distinguished from the gel using the “Cleanup Mini” set for purifying DNA from gel (“Eurogene” Ltd., Moscow, Russia). Sanger sequencing method was applied.

### 2.3. Editing and Alignment of Sequences

For editing and alignment of sequences, we used BioEdit v7.2.6 [15] and MEGA 7 programs [16]. Aroundbout 100–150 bp were poorly obtained at the beginning and end of the request. We deleted them. After checking the quality of sequences, for the further work, we took a fragment with a length of 721 bp, which was located in the range of 138–858 bp according to the NC_000845.1 reference sequence.

### 2.4. Genetic Diversity

To assess genetic diversity, we determined the number of haplotypes (H), haplotypic (Hd) and nucleotide (Pi) diversity, and the average number of nucleotide substitutions per site (K) using the DnaSP 5.10 program [17]. The synthesized score (Fz) was calculated by analyzing the main PCA components [18].

### 2.5. Haplogroups of MtDNA

Calculations and construction of trees using the ML (maximum likelihood) method were performed in the MEGA 7.0 program [16]. Haplogroup affiliation was determined in accordance with the sequences of the NCBI base: haplogroups A (GenBank: KT279758), B (GenBank: KT261429), C (GenBank: KT279759), D (GenBank: KT279760), and E (GenBank: KT261430).

### 2.6. Differences in the Distribution of Haplotypes and Haplogroups of MtDNA

To assess the significance of differences in the distribution of haplotypes and haplogroups of mtDNA, we used the two-tailed Fisher’s exact test calculated by the “fisher.test” command in the R-studio system [19].

## 3. Results

### 3.1. Genetic Diversity of Large White and Yorkshire Pig Populations in Russia

We studied 402 sequences of the D-loop fragment isolated from pigs kept in Russia from 2000 to 2020. The genetic diversity indicators of the Hd, Pi, and K of investigated pigs (Table 2) were analyzed by using correlation analysis and PCA. All three parameters were positively correlated and the degree of correlation Pi and K was 1.0 (Table 3).

### 3.2. Polymorphic Sites, Haplotypes, and Haplogroups

**Figure 1:**
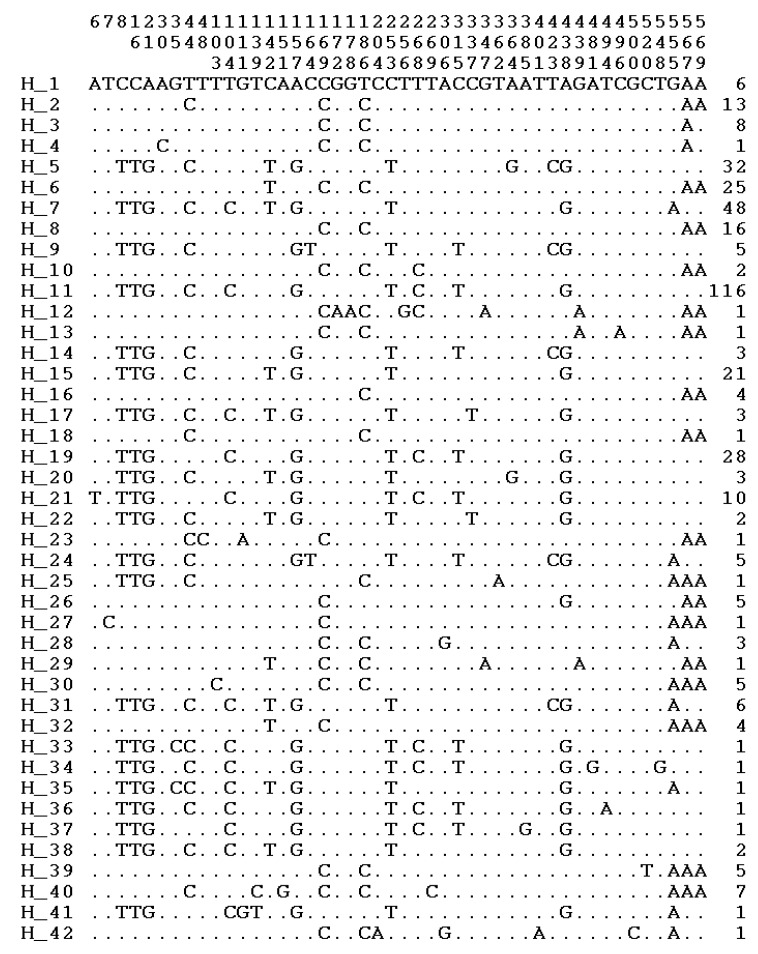
Polymorphic sites and haplotypes.

**Figure 2:**
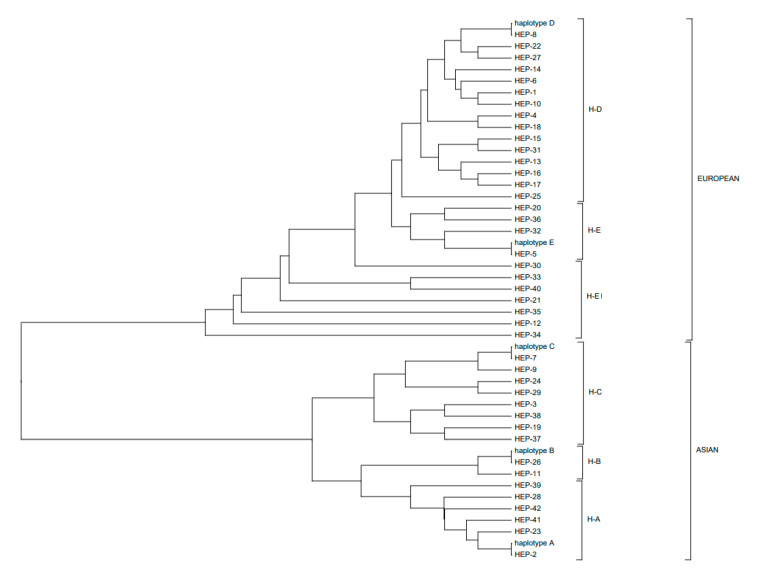
Distribution of haplotypes relative to haplogroups A, B, C, D, E.

In the studied sample, we defined 46 polymorphic sites and 42 haplotypes (Figure 1). The table with haplotype pigs is presented in Supplementary Materials. The haplotype distribution by clusters corresponding to the Asian (A, B, C) and European (D, E) haplogroups is shown in Figure 2. Construction of the phylogenetic tree by the maximum likelihood method showed that the haplotypes belonged to haplogroups A, B, C, D, E, and we highlighted a separate cluster near haplogroup E, which we contingently named E1.

Hap_11, determined in 28.9% of pigs (*n* = 116), had the highest frequency in the studied sample, while in the Old group, its frequency was 21.8% (*n* = 17), in Imp, 19.5% (*n* = 24), and in New, 31.3% (*n* = 75). High frequency (11.9%, *n* = 48) and representation in three groups (Old, 12.8%, *n* = 10; Imp, 7.3%, *n* = 9; New, 14.4%, *n* = 29) was defined for Hap_7. In total, only four haplotypes were present in all groups—these are the abovementioned Hap_11 and Hap_7, as well as Hap_1 and Hap_8.

### 3.3. Haplotype and Haplogroup Frequencies between the Old, Imp, and New Groups of Pigs

Despite the presence of common haplotypes, there were haplotypes found only in one of the groups. When comparing haplotype frequencies between the Old, Imp, and New groups, significant differences were found (Table 5).

**Figure 3:**
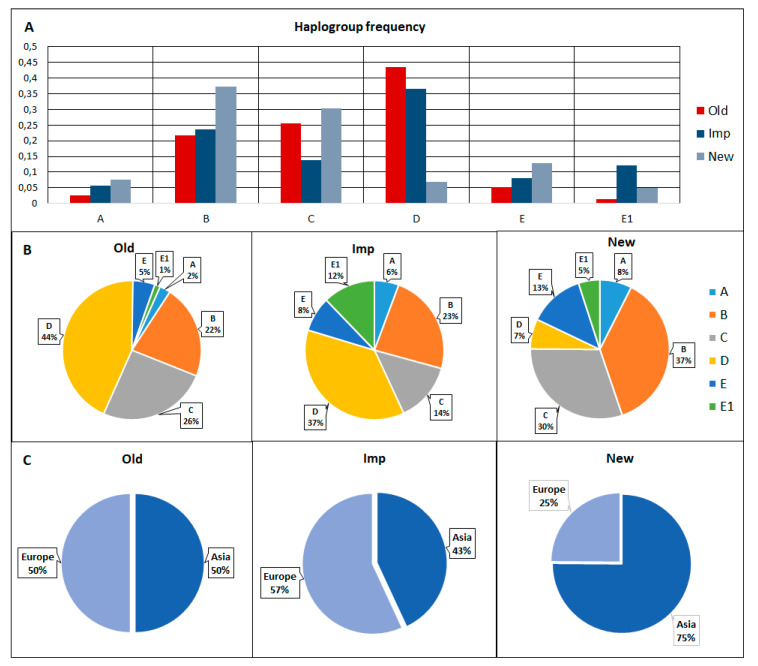
Frequencies of mtDNA haplogroups in Old, Imp, and New pigs (**A**—frequency distribution of haplogroups A, B, C, D, E and E1 in pigs of groups Old, Imp and New; **B**—frequency distribution of haplogroups A, B, C, D, E and E1 in each group; **C**—frequency distribution of haplogroups Asia (A, B, C) and Europe (D, E, E1) in pigs of groups Old, Imp, and New).

Therefore, the Нар_12 haplotype was found only in LW_2_Old, Нар_13, and Hap_14 in LW_4_Old. In the Imp group, we identified 11 unique haplotypes, five of which (Нар_18, 23, 25, 27, 29) were identified in only one animal and six (Hap_20–22, 26, 30.31) in more than one animal but also only on one farm. In the New group, 11 unique haplotypes were found, seven of which (Нар_33–37, 41, 42) were found in only one animal and four (Hap_32, 38–40) in more than one animal. Figure 3 shows the frequency distribution of mtDNA haplotypes and haplogroups in pigs of the Old, Imp, and New groups.

The distribution of haplogroups also differed between the Old, Imp, and New groups. In the Old group, haplotype D had the highest frequency, and, in general, the distribution for Asian and European haplotypes was 50/50. European haplogroups prevailed in the Imp group (*n* = 70; 56.9%) and haplogroup D had the highest frequency. Asian haplogroups prevailed in the New group (*n* = 151; 75%) and group B haplotypes had the highest frequency.

### 3.4. Haplotypes and Haplogroups in Pigs of the New Group

**Figure 4:**
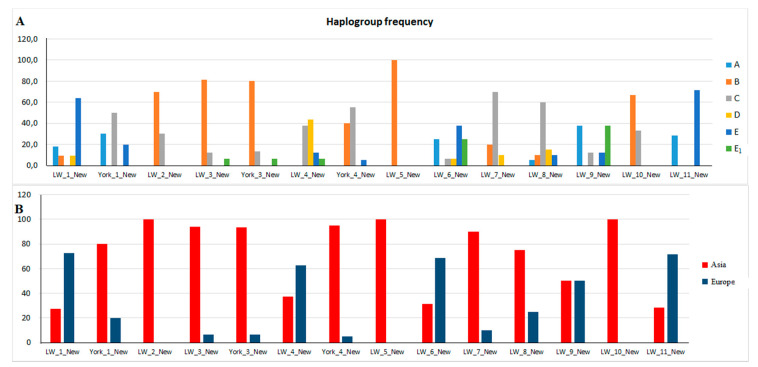
Frequencies of mtDNA haplogroups in New pigs (**A**—frequency distribution of haplogroups A, B, C, D, E and E1 in the pigs of the group New from different farms; **B**—frequency distribution of haplogroups Asia (A, B, C) and Europe (D, E, E1) in the pigs of the group New from different farms).

The diversity of haplotypes and haplogroups in pigs of the New group was associated with the farms in which they were bred (Figure 4). Despite the fact that Asian haplotypes prevailed in this group, it is possible to distinguish farms (LW_1, LW_4, LW_6, LW_9, and LW_11) where European haplotypes constitute 50% or more.

### 3.5. Frequencies of Haplotypes and Haplogroups in Old_Center, Old_Siberia, and Old_South Groups of Pigs

**Figure 5:**
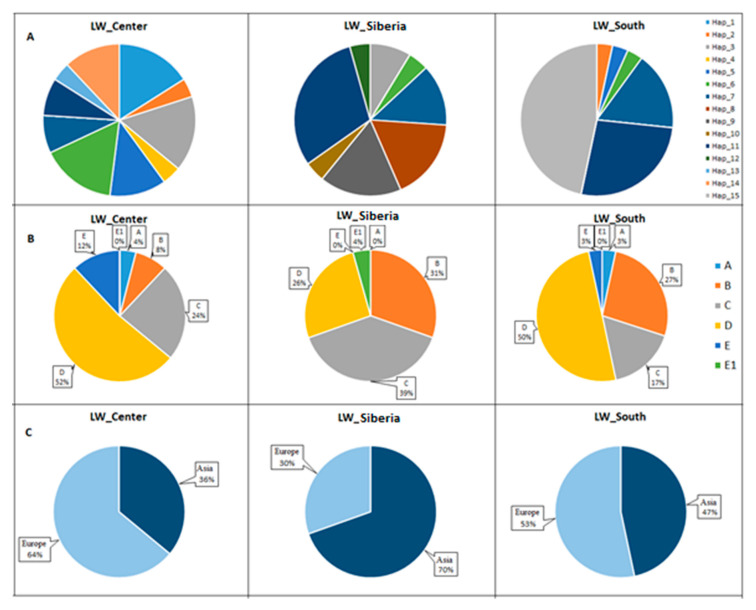
Frequencies of mtDNA haplotypes and haplogroups in the LW_Old group (**A**—frequency distribution of haplotypes in pigs of clusters Center, Siberia and South; **B**—frequency distribution of haplogroups A, B, C, D, E and E1 in pigs of clusters Center, Siberia and South; **C**—frequency distribution of haplogroups Asia (A, B, C) and Europe (D, E, E1) in pigs of clusters Center, Siberia and South).

Furthermore, according to stated objectives, we found that, in the Old group, the frequency distribution of haplotypes was associated with pigs’ adaptation to different climatic conditions. To evaluate this, the pigs of the Old group were divided into three clusters corresponding to the Southern region of the Russian Federation (South), the Central region of the Russian Federation (Center) and Siberia (Siberia). In total, we identified 15 haplotypes in the Old group pigs, which were distributed among Asian (A, B, C) and European (D, E, E1) haplotypes (Figure 5).

The results showed that haplotype frequencies significantly differed between the Old_Center, Old_Siberia, and Old_South clusters (Table 6).

Significant differences in the haplogroup frequencies were defined only between the Old_Center and Old_Siberia groups. In the Old_Center pigs, haplogroup D prevailed (52%), while in the Old_Siberia, haplogroup C dominated (39%). Altogether, 70% of the Old_Siberia pigs had Asian haplogroups, compared to the Old_Center pigs with 64% of European haplogroups. In the Old_South cluster, the distribution for Asian and European haplogroups was 47% and 53%, respectively.

## 4. Discussion

Mitochondria are responsible for 95% of the energy of a eukaryotic cell through oxidative phosphorylation of ADP (adenosine diphosphate) forming ATP (adenosine triphosphate). Thus, some variations in mtDNA can have important consequences for animals when adapting to different environmental conditions [20,21,22]. Evidence of adaptive evolution affecting mtDNA is presented by Niu et al. [23], Slimen et al. [24], Xu et al. [25], and others.

In our study, we chose Large White pigs, which were selected after long-term targeted selection work for pig farming in Southern region, Central region and Siberia. For Southern region, dry and very hot summers and mild winters are inherent (the temperature can reach 35—40 °C); for the Central region, mildly cold winters and humid, moderately warm summers; for Siberia, cold winters with temperature reaching 40 °C and moderate–warm summers. Due to the fact that pigs of the Old group were distinguished by good productivity despite very high summer temperatures (Old_South) and quite severe winters (Old_Siberia), we suggested that this might be connected with mtDNA haplotypes. 

Our findings do not fully prove that any genotypes are more or less preferable for different climatic conditions. However, they show local evidence that the distribution of haplotype frequencies is related to the geographical locations of zones with different climatic conditions and can be the basis for further research connected with searching loci of supposedly neutral and adaptive components of genetic diversity in mitochondrial and nuclear DNA.

Despite the high adaptation and good productivity of local pigs in the late XX–early XXI centuries, Russia began to import pigs of other selections which were significantly superior in growth rate and reproductive performance. The import pigs were too exacting to keep and feed, but despite this, they quickly replaced the local.

Our results showed that Large White pigs of the Old group had both European and Asian haplotypes. In 2008–2010, Large White pigs imported to the Russian Federation had predominantly European haplotypes. However, currently, in Large White pigs (from leading genetic centers), Asian haplotypes prevail. Perhaps this is due to targeted breeding aimed at increasing productivity, including sow fertility [26]. In European pig farming, we can observe a steady increase in litter size during the last three decades [27]. Currently, litters can reach up to 18–20 piglets [26,28]. To some extent, this is due to the introduction of Chinese genes to commercial European breeds [29,30].

Chinese breeds are highly fertile. The role of the mitochondrial genome in this process is still not clear today. Mitochondrial diversity has long been considered neutral or almost neutral [31]. However, an increasing amount of research with animal models and humans suggests that mtDNA variants may be associated with some phenotypic traits [32,33,34,35,36,37,38]. Given this, we can assume that further intensification of pig production can lead to a significant spread of some haplotypes and disappearance of others.

Over the past decade, the global commercial pig breeding industry has provoked significant business consolidation. The mergers and acquisitions of small genetic centers has led to a small number of remaining internationally operating breeding companies [39]. Hence, the breeding lines belonging to these companies also have undergone a high degree of consolidation. Each of these companies uses its own selection technologies and target indices while improving the breeds and this in its turn makes a significant difference in the breed genetic structure.

Regarding Large White pigs raised in Russia, we can note that, alongside consolidating enough livestock in separate farms, other farms demonstrate high genetical diversity, with some farms having mainly European haplotypes and others, on the contrary, having Asian ones. Genetic variation in Large White pigs inside the farms may depend on the strategy of the economy, preferring either a rigid consolidated frame (in the case of working only with a certain selection center) or a more variable one allowing farmers to variate and improve their livestock.

## 5. Conclusions

The results of studying the mtDNA of pigs have shown the intrapedigree genetical diversity of the Large White breed. Peculiar properties of the genetical structure of mtDNA are bound to the period of their breeding in Russia; particularly, it is seen in Large White pigs of the Russian selection and the modern (commercial) livestock of the Large White breed. Along with this, we can trace certain distinctions of mtDNA haplotypes and haplogroups caused by differences in farming in the Russian Federation. This directly is bound to the selection strategy of international genetic centers where, in spite of significant consolidation of genetical structure inside the center, a significant general genetical diversity of the breed is provided. Besides the above, the obtained results indicate a connection between the frequency distribution of mtDNA haplotypes and adaptation to various climatic conditions in Large White pigs of the Russian selection.

As a whole, the presented results are an impetus for further investigations of mtDNA, and, with consideration of the functional importance of certain peptides encoded by mitochondrial genes, they introduce an interest in understanding the processes which are stimulating and sustaining variations in mtDNA.

## Figures and Tables

**Table 1:** Pigs under study.

Group	Farm	*n*	Year of Sampling	Place of Sampling
Old, *n* = 78	LW_1_Old	12	2006	Central District (Center)
	LW_2_Old	12	2011	Siberia (Siberia)
	LW_3_Old	11	2005	Siberia (Siberia)
	LW_4_Old	13	2003	Central District (Center)
	LW_5_Old	16	2009	Southern District (South)
	LW_6_Old	14	2011	Southern District (South)
Imp, *n* = 123	York_1_Imp	20	2008	
	York_2_Imp	13	2010	
	York_3_Imp	16	2013	
	York_4_Imp	16	2010	
	York_5_Imp	15	2011	
	York_6_Imp	16	2014	
	LW_1_Imp	13	2011	
	LW_2_Imp	7	2010	
New, *n* = 201	LW_1_New	11	2017	
	LW_2_New	10	2018	
	LW_3_New	16	2017	
	LW_4_New	16	2018	
	LW_5_New	20	2018	
	LW_6_New	16	2018	
	LW_7_New	20	2018	
	LW_8_New	20	2017	
	LW_9_New	8	2017	
	LW_10_New	12	2018	
	LW_11_New	7	2018	
	York_1_New	10	2017	
	York_3_New	15	2018	
	York_4_New	20	2018	

In Table 1: For pigs of Imp and New groups, the place of sampling was not specified as they were kept in enclosed spaces with controllable climate conditions.

**Table 2:** Parameters of genetic diversity of Large White and Yorkshire pig populations in Russia.

Group and Farm	N	S	H	Hd	K	Pi
LW_1_Old	12	17	7	0.879	6.424	0.00891
LW_2_Old	12	23	8	0.894	9.500	0.01318
LW_3_Old	11	16	4	0.745	7.782	0.01079
LW_4_Old	13	20	7	0.897	8.615	0.01195
LW_5_Old	16	17	5	0.767	3.875	0.00537
LW_6_Old	14	16	4	0.582	3.066	0.00425
LW_1_Imp	20	18	5	0.763	8.058	0.01118
York_1_Imp	20	17	7	0.800	5.179	0.00718
LW_2_Imp	7	18	4	0.810	7.333	0.01017
York_2_Imp	13	18	3	0.410	4.179	0.00580
York_3_Imp	16	15	2	0.125	1.875	0.00260
York_4_Imp	16	18	8	0.875	5.483	0.00761
York_5_Imp	15	19	6	0.819	7.867	0.01091
York_6_Imp	16	18	4	0.742	8.183	0.01135
LW_1_New	11	18	5	0.818	8.145	0.01130
York_1_New	10	19	4	0.733	8.111	0.01125
LW_2_New	10	4	2	0.467	1.867	0.00259
LW_3_New	16	5	3	0.342	1.058	0.00147
York_3_New	15	6	3	0.362	1.257	0.00174
LW_4_New	16	17	5	0.792	5.542	0.00769
York_4_New	20	6	5	0.663	1.026	0.00142
LW_5_New	20	0	1	0.000	0.000	0.00000
LW_6_New	16	21	5	0.775	9.617	0.01334
LW_7_New	20	16	5	0.558	2.368	0.00328
LW_8_New	20	8	5	0.626	1.921	0.00266
LW_9_New	8	25	6	0.893	10.143	0.01407
LW_10_New	12	4	2	0.485	1.939	0.00269
LW_11_New	7	13	2	0.476	6.190	0.00859
Old	78	26	15	0.890	7.452	0.01034
Imp	123	29	25	0.915	8.442	0.01171
New	201	33	23	0.810	5.971	0.00828
ALL	402	46	42	0.881	7.431	0.01031

In Table: N—number of samples, S—number of substitutions, H—number of haplotypes, Hd—haplotype diversity, Pi—nucleotide diversity, K—average number of nucleotide substitutions per site.

**Table 3:** Correlation matrix between indexes.

Item	Hd	Pi	K
Hd	1.0	0.771	0.771
Pi	0.771	1.000	1.000
K	0.771	1.000	1.000

To assess genetic diversity, we extracted two components using the PCA method. F1 shows the changes in Pi and K; F2 shows the changes in Hd. Then, we calculated the synthesized score of Fz (Table 4).

**Table 4:** Scores and general PCA scores of different populations.

Breed	F1	F2	Fz
LW_1_Old	−0.469869	0.397603	−0.323178
LW_2_Old	−0.216537	−0.792395	−0.393317
LW_3_Old	−0.453041	0.412831	−0.304223
LW_4_Old	−0,410942	−0.075118	−0.388568
LW_5_Old	0.009080	0.883065	0.229365
LW_6_Old	0.593214	−0.192177	0.485617
LW_1_Imp	−0.687686	0.641848	−0.457983
York_1_Imp	0.458763	−0.774221	0.218848
LW_2_Imp	−0.852489	0.334876	−0.683160
York_2_Imp	1.216740	1.077880	1.364776
York_3_Imp	0.085042	−0.801193	−0.124170
York_4_Imp	0.866407	1.090529	1.052726
York_5_Imp	0.330318	0.614465	0.451124
York_6_Imp	−0.525864	0.103496	−0.447231
LW_1_New	−0.115446	−0.590256	−0.251726
York_1_New	−0.157773	−0.466608	−0.258837
LW_2_New	−0.651816	−0.072000	−0.604517
LW_3_New	−0.418594	0.283185	−0.305703
York_3_New	−0.345033	−0.566545	−0.452361
LW_4_New	0.548433	−1.142167	0.207365
York_4_New	0.453650	−0.404940	0.306747
LW_5_New	0.341198	−0.315651	0.227932
LW_6_New	−0.470192	0.287218	−0.351119
LW_7_New	0.801264	0.068671	0.738151
LW_8_New	0.569170	−0.241109	0.451726
LW_9_New	−0.489366	0.028054	−0.433288
LW_10_New	−0.767598	0.166413	−0.648975
LW_11_New	0.758969	0.044248	0.693978

**Table 5:** Two-tailed Fisher’s exact test in groups of Old, Imp, and New.

Clusters	Two-Tailed Fisher’s Exact Test		
	Haplotype	Haplogroup	Asia/Europe
Old-Imp	0.0005 ***	0.0225 *	0.3843 ***
Old-New	0.0005 ***	0.0005 ***	9.521 × 10^−5^ ***
Imp-New	0.0005 ***	0.0005 ***	9.446 ×10^−9^ ***

*—*p* ≤ 0.05; ***—*p* ≤ 0.001.

**Table 6:** Two-tailed Fisher’s exact test in Old group clusters.

Clusters	Two-Tailed Fisher’s Exact Test		
	Haplotype	Haplogroup	Asia/Europe
Old_Center—Old_Siberia	0.0010 ***	0.0270 *	0.0247 *
Old_Center—Old_South	0.0005 ***	0.3348	0.5837
Old_Siberia—Old_South	0.0001 ***	0.1439	0.1615

*—*p* ≤ 0.05; ***—*p* ≤ 0.001.

## Supplementary Materials

The following are available online at https://www.mdpi.com/2076-2615/10/8/1365/s1, Table S1: Haplotypes in the studied group of pigs.
